# In Silico Analysis of Anti-Inflammatory and Antioxidant Properties of Bioactive Compounds from *Crescentia cujete* L.

**DOI:** 10.3390/molecules28083547

**Published:** 2023-04-18

**Authors:** Alecsanndra L. Gonzales, Steven Kuan-Hua Huang, Ureah Thea A. Sevilla, Cheng-Yang Hsieh, Po-Wei Tsai

**Affiliations:** 1 School of Chemical, Biological, Materials Engineering and Sciences, Mapúa University, Manila 1002, Philippines; algonzales@mymail.mapua.edu.ph (A.L.G.); utasevilla@mapua.edu.ph (U.T.A.S.); 2 Department of Medical Science Industries, College of Health Sciences, Chang Jung Christian University, Tainan 711, Taiwan; 7224837@mail.cjcu.edu.tw; 3 Division of Urology, Department of Surgery, Chi Mei Medical Center, Tainan 711, Taiwan; 4 School of Medicine, College of Medicine, Kaohsiung Medical University, Kaohsiung 807, Taiwan; 5 Ph.D. Program in Clinical Drug Development of Herbal Medicine, College of Pharmacy, Taipei Medical University, Taipei 110, Taiwan; 6 Laboratory of Oncology Pharmacy Practice and Science, Graduate School of Pharmaceutical Sciences, Tohoku University, Sendai-shi 980-8577, Japan

**Keywords:** *Crescentia cujete*, antioxidant, molecular docking, flavonoids, ADME prediction, anti-inflammatory

## Abstract

*Crescentia cujete* is widely known as a medical plant with broad indigenous ethnomedicinal uses, including anti-inflammatory, and antioxidant. Despite being used for remedies and ethnomedicinal purposes, the benefits obtained from *C. cujete* still need to be fully utilized. The underwhelming studies on its pharmacological potential, bioactive compounds, and mechanism of action keep the pharmacological and new drug discovery progress of this plant slow. This study focuses on the incorporation of in silico analyses such as ADME prediction and molecular docking simulations on the bioactive compounds identified in the plant to assess their potential for antioxidant and anti-inflammatory applications. A comparison of the ADME properties and molecular docking scores showed that naringenin, pinocembrin, and eriodictyol had the most potential to act as inhibitors of the target proteins involved in inflammation and oxidation pathways against the positive controls.

## 1. Introduction

Inflammation-mediated responses in the body are associated with many diseases, such as asthma, chronic inflammatory diseases, diabetes, cancer, and atherosclerosis. In particular, chronic inflammation sets off a series of disease-causing effects, and people with this have a much greater risk of developing a serious condition. Inflammation has been recognized as one of the major causes of disease. It is estimated that 15% of human cancers are associated with chronic infection and inflammation [1].

*Crescentia cujete*, also known as Calabash, belongs to the Bignoniaceae family. Its fruit has seeds and a white pulp that, over time, changes into black, which is of medicinal value. Other parts of the plant and fruit are all useful in several ways: the hard and empty shells are used for storing foodstuff and can also be used in crafts and arts, while the leaves are often brewed by locals and drunk as a medicinal tea. Its wood is also used to make tools such as utensils and boats. Previous studies focused on exploring the bioactive components of *C. cujete* show that it contains minerals such as calcium, magnesium, and sodium, along with phytochemicals such as flavonoids, phenols, saponins, alkaloids, and tannins [2].

Flavonoids are polyphenolic compounds known for their antioxidant property which helps to protect cells from free radical damage and prevent the rise of various health problems [3]. They are also known to help maintain healthy circulation and are anti-inflammatory, anti-viral, and capillary strengthening. Phenols are a group of compounds that are commonly used in disinfection and the standard used to compare bactericides [4]. Plants with phenols are proven to help ward off infection or insect attacks. Phenols also have antibacterial, antioxidant, anti-inflammatory, antiseptic, and anti-viral properties [5]. Similar to most medicinal plants, calabash also contains saponins, a natural antibiotic and energy booster. They also help to reduce inflammation of the upper respiratory tract, reduce body cholesterol by preventing its re-absorption, and suppressing rumen protozoa by dissolving it through its reaction with cholesterol in the protozoan cell membrane [6]. Alkaloids are also known for their anti-inflammatory property and their analgesic, antispasmodic, and bactericidal properties. While tannins are known for their astringent properties, which are applied in wound healing, tightening up loose tissues, and drying up secretions, they are also used for treating urinary tract infections (UTI) and other bacterial infections due to their ability to prevent decay and their antimicrobial activity [2,7].

Although some studies are exploring the bioactive components of *C. cujete*, there were few that have focused on its actual medical application. All studies about this plant prove that it contains phytochemicals and bioactive compounds that can be beneficial in making advances in the biopharmaceutical sense. So far, no study has focused on this plant’s mechanism of action in terms of its antioxidant, anti-inflammatory, antimicrobial, and anti-fungal properties on the molecular level, particularly at the enzyme level [8].

Knowing the phytochemicals and bioactive compounds in *C. cujete* is insufficient to explore and optimize its medical value. This study aims to fill the gaps from the previous studies by producing an aqueous, semi-purified extract from *C. cujete* and performing bioassays along with in vivo analysis to prove that the extract obtained from *C. cujete* is a potential drug candidate for treating patients with inflammatory-related diseases and for limiting free radical damage [9].

This study observes the following pharmacological properties of *C. cujete*: anti-inflammatory and antioxidant, through in silico studies, focusing on phytochemicals including flavonoids, phenols, and saponins. Molecular docking simulations and ADMET predictions were conducted to observe the phytochemical and protein–ligand complex with the most optimal anti-inflammatory and antioxidant properties. The use of basic metabolomics combined with molecular docking and simulation provides a deeper understanding of how the secondary metabolites present in the *C. cujete* plant works to provide potential future research ideas for pharmacological applications and drug-development advancements.

## 2. Results

### 2.1. ADME Prediction

To estimate the individual ADME (absorption, distribution, metabolism, and excretion) behaviors of the chosen compounds from the plant is essential in the initial stage of the drug discovery or development processes.

The bioavailability radar enables a glimpse of a molecule’s drug-likeliness with the pink-highlighted area as the optimal range for each property: FLEX for the flexibility of the molecule as per rotatable bonds (≤9 bonds), SIZE for the size as the molecular weight (between 150–500 g/mol), POLAR for polarity as per the topological polar surface area (between 20–130 Å2), INSOLU for insolubility in water by log S (≤6), and INSATU for saturation as per fraction of carbons in the sp3 hybridization (≥0.25) [10].

**Figure 1:**
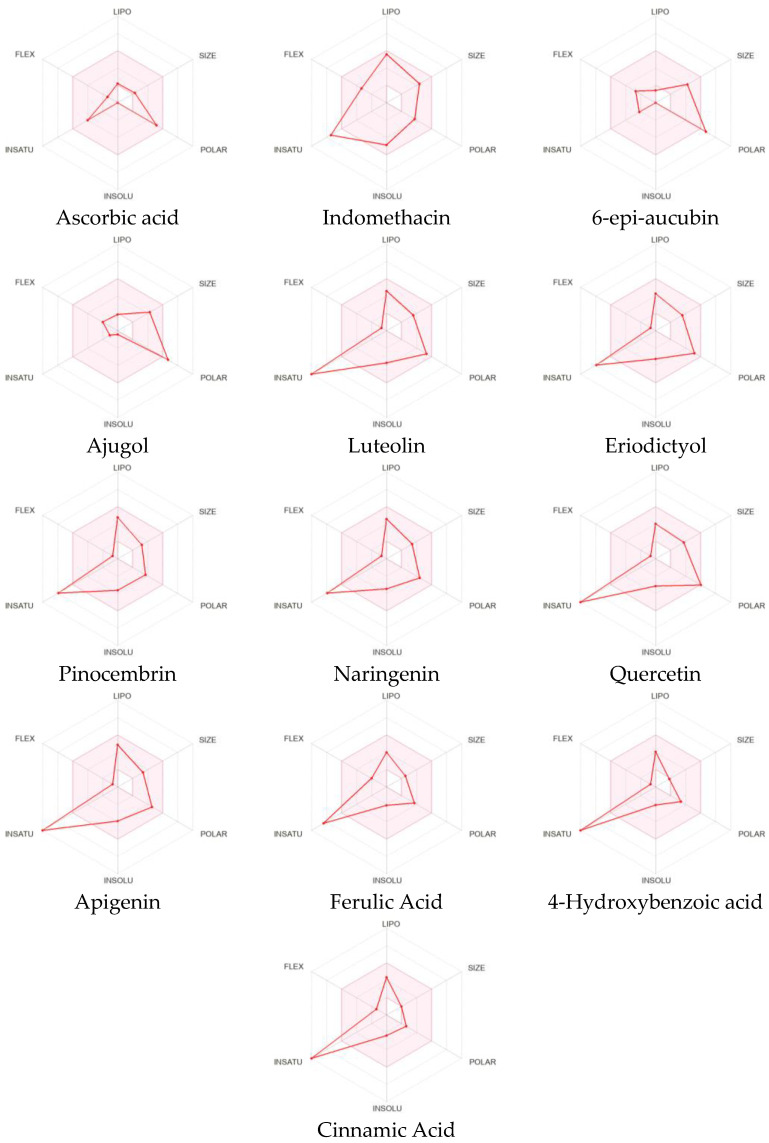
SwissADME bioavailability radar for drug-likeness of all identified compounds from *Crescentia cujete*, indomethacin and ascorbic acid.

Comparing the bioavailability radar of all of the compounds from *C. cujete* and the positive controls (ascorbic acid and indomethacin) (Figure 1), only the ascorbic acid has all properties fitting within the optimal range making it the only compound predicted to be orally bioavailable.

### 2.2. Molecular Docking

**Figure 2:**
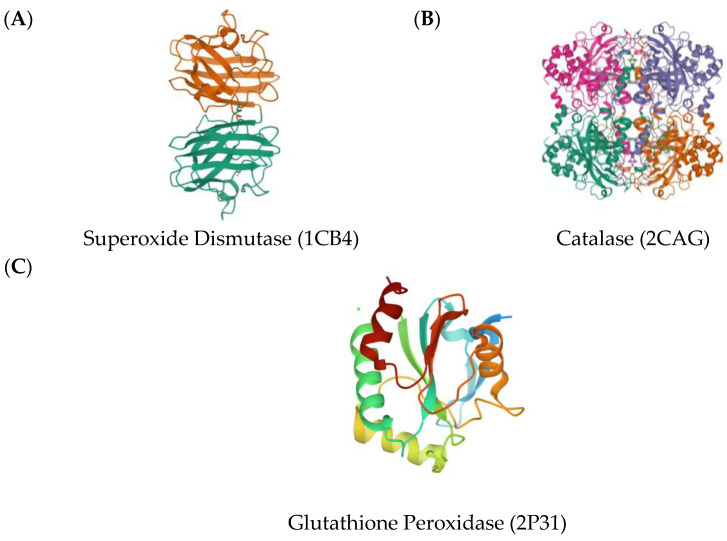
Three-dimensional crystal structures of Copper, (**A**) Zinc Superoxide Dismutase (1CB4); (**B**) Catalase compound II (2CAG); (**C**) Human Glutathione Peroxidase 7 (2P31).

As explored in previous studies, a lot of phytochemicals act as agonists of superoxide dismutase (SOD) (Figure 2A), glutathione peroxidase (GPX) (Figure 2B), and catalase (CAT) (Figure 2C), increasing their activity [11]. An increase in the activity of any of these proteins helps to overcome the stress related to ROS reduction. Hence, these proteins were used as the target proteins to simulate the antioxidant activity of the phytochemicals (Figure 2).

**Figure 3:**
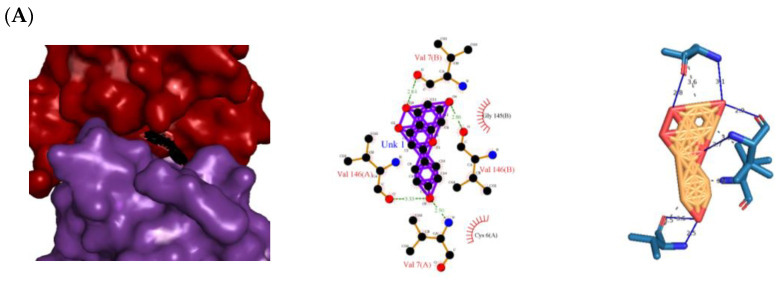

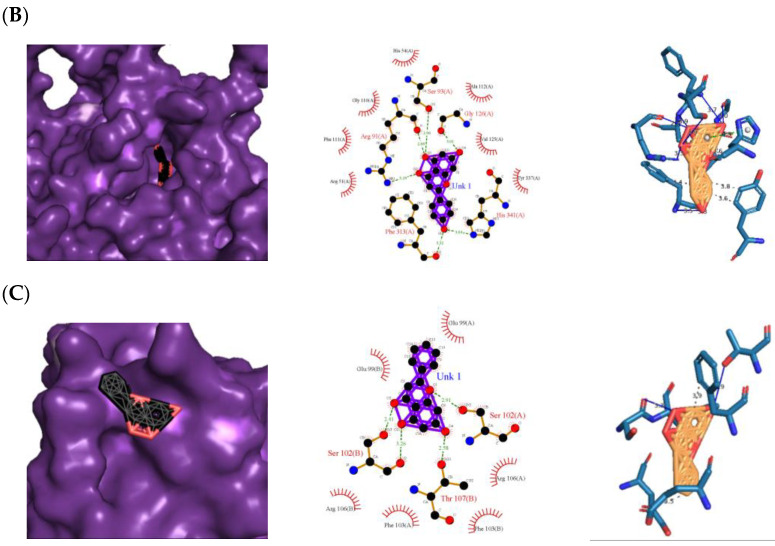
Top protein–ligand complex 3D structures, 2D and 3D ligand–protein interaction diagrams from LigPlot for antioxidant activity. (**A**) 1CB4-naringenin complex; (**B**) 2CAG-naringenin complex; (**C**) 2P31-pinocembrin complex.

The 3D and 2D diagrams of the top ligand docked on the active site for each protein are shown in Figure 3. Naringenin’s atoms contributed the following H bonds with the backbones of 1CB4/2CAG (Figure 3A,B): One from the O_2_ atom with the backbone of Arg 91(A) in 2CAG; three from the O_3_ atom with Val 7(B)/Ser 93(A) and Arg 91(A); two from the O_4_ atom with Val 146(B)/Gly 126(A); and four from the O_5_ atom with Val 7(A),Val 146(A)/Phe 313(A), and His 341(A). H bonds were considered to be the facilitators of the protein–ligand binding as they displace protein-bound water molecules and facilitate the free-energy barrier reduction during enzyme catalysis [12].

Besides the H bonding, naringenin also hydrophobically interacted with the following amino acid residues: Gly 145(B), Cys 6(A), Val 146(A and B), Val 7(A and B) for 1CB4 protein, Gly 110(A), His 54(A), Phe 111(A), Arg 51(A), Ala 112(A), Val 125(A), and Tyr 337(A) for 2CAG protein (Figure 3B). Hydrophobic interactions are essential for protein–ligand complexes as they enhance the binding affinity and biological activities of the complex molecules, which helps to stabilize their biochemical environment [13]. The presence and number of the H bonds and hydrophobic interactions of naringenin with the proteins 1CB4 and 2CAG support the findings obtained from the molecular docking simulation, which indicates that naringenin has the potential to be a dual agonist for 1CB4 and 2CAG proteins with a binding energy of −10.32 kcal mol^−1^ and −11.87 kcal mol^−1^, respectively, compared to the standard used, which is ascorbic acid (Table 1).

All chosen flavonoids scored significantly higher than the control (ascorbic acid) when docked with all three proteins having eriodyctiol consistently being the third best-scored ligand, naringenin being the top-scored when docked with superoxide dismutase protein (1CB4) and catalase compound II protein (2CAG), and pinocembrin being the top-scored when docked with the human glutathione peroxidase 7 protein (2P31) (Figure 3C).

**Figure 4:**
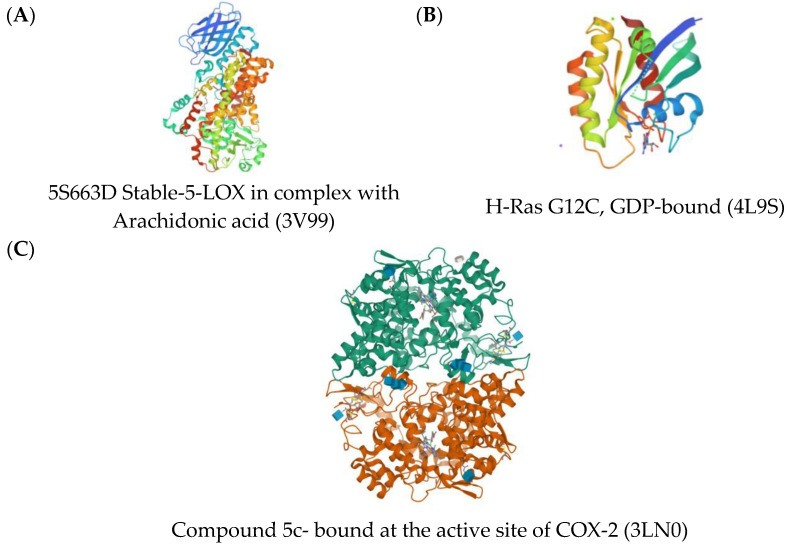
Three-dimensional crystal structures of (**A**) 5S663D Stable-5-LOX in complex with Arachidonic acid (3V99); (**B**) H-Ras G12C, GDP-bound (4L9S); (**C**) compound 5c-S bound at the active site of COX-2 (3LN0).

Cyclooxygenase (COX), lipoxygenase (LOX), and H-Ras signaling proteins are some of the proteins directly affecting inflammation; hence, these proteins were used as the target proteins for the simulation of the anti-inflammatory activity of the flavonoids Figure 4).

**Figure 5:**
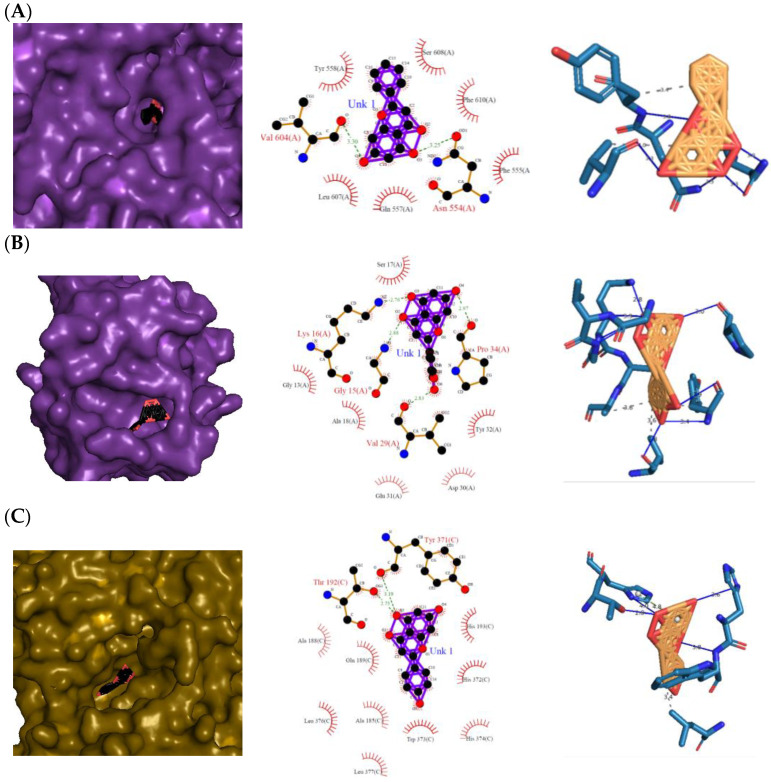
Top protein–ligand complex 3D structures, 2D and 3D ligand–protein interaction diagrams from LigPlot for anti-inflammatory properties. (**A**) 3V99–pinocembrin complex; (**B**) 4L9S–eriodictyol complex; (**C**) 3LN0–naringenin complex.

The 3D and 2D diagrams of the top ligand docked on the active site for each protein are shown in Figure 5. Pinocembrin’s O_3_ and O_4_ atoms only contributed to two H bonds with the backbones of the LOX-5 protein (3V99) (Figure 5A): Asn 554(A) and Val 604(A), respectively. However, pinocembrin hydrophobically interacted with six amino acid residues: Tyr 558(A), Ser 608(A), Phe 610(A), Phe 555(A), Gln 557(A), and Leu 607(A), causing a free-binding energy of −11.29 kcal mol^−1^ (Table 2), despite the complex having only two H bonds. On the other hand, the following atoms of eriodictyol contributed to H bonds with the backbones of H-Ras signaling protein (4L9S) (Figure 5B): one from the O_2_ atom with Gly 15(A), one from the O_3_ atom with Lys 16(A), one from the O_4_ atom with Pro 34(A), and one from the O_6_ atom with Val 29(A), while the ligand hydrophobically interacted with the Ser 17(A), Gly 13(A), Ala 19(A), Glu 31(A), Tyr 32(A), and Asp 30(A) of the protein. For the COX-2 protein (3LN0) (Figure 5C), naringenin was found to be the top-scored ligand with a −11.96 kcal mol^−1^ atom O_3_ contributing to two H bonds with Tyr 371(C) and Thr 192(C), and hydrophobic interactions with Ala 188(C), Glu 189(C), Leu 376(C), Ala 185(C), Leu 377(C), Trp 373(C), His 374(C), His 372(C), and His 193(C). Arginine, tryptophan, leucine, and alanine were some of the precursors for an innate inflammatory response while starvation or reduction of reserved amino acids such as tyrosine, histidine, and glutamine on signaling proteins activated certain downstream pathways and upregulation of autophagy which results in the restriction of inflammation [14].

## 3. Materials and Methods

### 3.1. ADME Prediction

ADME property prediction. The selected compounds’ ADME properties (i.e., absorption, distribution, metabolism, excretion, and toxicity) were predicted using Molinspiration, the SwissADME predictor, and pKCSM pharmacokinetics [15]. The following data were gathered: the numbers of hydrogen donors and acceptors, rotatable bonds, total polar surface area, and their synthetic accessibility after subjecting the pharmacological compounds to ADME analysis based on the five rules described in Lipinski et al. [16], Muegge et al. [17], Ghose et al. [18], Egan et al. [19] and Veber et al. [20]. Comparison with Indomethacin as the positive control was carried out for analysis, and only the compounds that did not violate any of the screenings were used for the molecular docking analysis [21].

### 3.2. Molecular Docking

To observe the anti-inflammatory and antioxidant activities of the chosen flavonoids from *C. cujete*., Autodock Tools and MGL tools were used and loosely based on a previous study conducted by Peasari et al. (2017) [22], partnered with LigPlot+ to simulate the 3D and 2D interactions of the ligand–protein complexes.

### 3.3. Retrieval of Protein Structure

Using the X-ray crystal structure of proteins, 5S663D Stable-5-LOX in complex with Arachidonic acid (PDB ID: 3V99) at 2.25 Ao resolution, H-Ras G12C, GDP-bound signaling protein (PDB ID: 4L9S) at 1.61 Ao resolution [23], and compound 5c-S bound at the active site of COX-2 (PDB ID: 3LN0) at 2.67 Ao resolution [24], retrieved from the RCSB Protein Data bank, the major role in oxidation and proliferation pathways was observed. Using the X-ray crystal structure of proteins Copper, Zinc Superoxide Dismutase (PDB ID45: 1CB4), Catalase compound II (PDB ID: 2CAG) and Human Glutathione Peroxidase 7 (PDB ID: 2P31), all at 0.30 Ao resolution [11], retrieved from the RCSB Protein Data bank, the major role in inflammation and proliferation pathways was observed. All proteins were prepared for molecular docking by removing all water and other hetero molecules from the original structure. Active site analysis was also performed through the Swiss Protein Viewer SPDBV [25].

### 3.4. Retrieval of Ligands

The 3D structures of the flavonoids identified were retrieved from NCBI Pub Chem Compounds in SDF format and their 2D structures were then sketched using Chemspider and Molinspration.

### 3.5. Grid Preparation and Molecular Docking

Using Autodock Tools [26] and MGL tools [27,28], docking was performed between selected macromolecules and ligands in which addition of hydrogen atoms and Kollman charges was carried out to prepare the protein molecules.

The ligand was prepared by setting flexible torsion angles at all rotatable bonds, while keeping the protein as a rigid structure. The Lamarckian Genetic Algorithm (LGA), which is a local search algorithm, was used to search for ligand conformations. Using the Autodock tool, the intermolecular energy (Kcal/mol) and inhibition constant (µN) of the docked complex forming hydrogen bonds and other parameters were analyzed. The ten best poses were generated for each ligand and scored using the local scoring functions, which was the basis for the ligands to be ranked. Schrodinger Suite was used to analyze the interacting residues of the ligands.

## 4. Discussion

The studies performed so far with regards to the plant *Crescentia cujete* only focused on in vitro assays of the plant’s crude extracts or fractionated extracts with Rivera-Mondragón’s study being an exception for isolating and identifying a total of sixty-six (66) bioactive compounds through UPLC-MS/MS-based profiling [29]. However, despite being previously identified, little to no data were found in terms of the ligand–residue interactions and ADME properties of the flavonoids identified from the plant (Table 3)—excluding quercetin which is one of the most studied flavonoids—with the proteins used as targets for the antioxidant and anti-inflammatory properties analyses, limiting the knowledge available.

**Figure 6:**
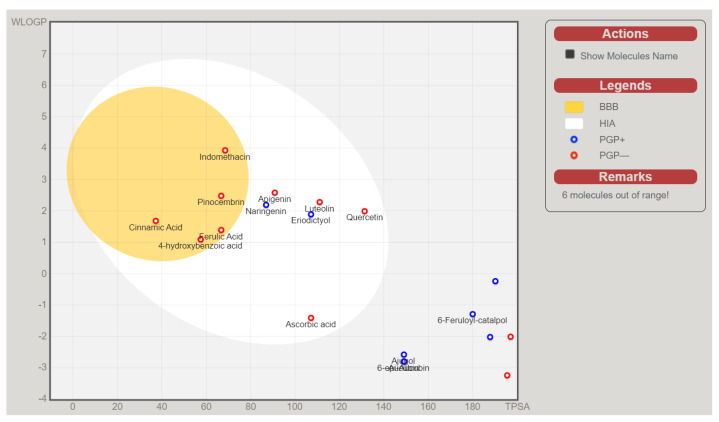
SwissADME BOILED–Egg diagram of the bioactive compounds from *Crescentia cujete* and positive controls Indomethacin (anti-inflammatory) and Ascorbic acid (antioxidant) for perceptive evaluation of their passive gastrointestinal absorption (HIA) and blood-brain barrier (BBB) penetration.

The above-presented data indicate that naringenin, pinocembrin, and eriodictyol are potential inhibitors of the inflammation and oxidation pathways’ target proteins. Without the high-HIA compounds (Figure 6), the rest of the identified compounds from *C. cujete* fell out of the BBB and HIA range, predicting their inability for absorption and brain penetration which was also the basis of these compounds to be disregarded for the succeeding in silico analysis. It was also observed that among the compounds with a high probability of passive absorption and brain penetration, only the eriodictyol and naringenin were predicted as actively effluxed by P-gp (PGP+) while the rest were predicted as non-substrates of P-gp (PGP−) [30].

Through the bioavailability radar result, it can be seen that the indomethacin and the rest of the compounds have their SIZE and POLAR (Supplementary Table S1), LIPO and INSOLU (Supplementary Table S2) properties within the optimal range, and they all fell out of the optimal range for saturation making them non-bioavailable. Additionally ajugol and 6-epi-aucubin were found to be a bit too polar to be considered orally bioavailable. Considering the pharmacokinetic properties of the compounds from *C. cujete*, most of the flavonoids exhibited the most promising properties with Quercetin leading with the highest skin permeability of −7.05 cm/s. This is followed by Eriodictyol with −6.62 cm/s and Ferulic acid with −6.41 cm/s, and has the highest probability to be passively absorbed through the gastrointestinal tract and penetrate the brain barrier without inhibiting any cytochromes (Supplementary Table S3). These flavonoids also showed the most promising “drug-likeness” having violated no rule (Lipinski, Ghose, Veber, Egan, and Muegge) and a bioavailability score of 0.55 each, excluding the ferulic acid with one Muegge violation and a higher bioavailability score of 0.85 (Supplementary Table S4). Only the same flavonoids also exhibited great medical chemistry with little to no PAINS and Brenk alerts, and lead-likeness violations except for Ferulic acid (Supplementary Table S5).

The insignificant number of alerts or violations supports the prediction that the majority of these compounds have no significant substructures showing a potent response in assays irrespective of the target protein, avoiding false positive biological outputs, and these compounds have stable metabolism, less reactiveness to chemicals, and better pharmacokinetics, which is consistent with previous studies on flavonoids [31]. However, with one PAINS and Brenk alert each, it is predicted that luteolin, eriodictyol, and quercetin have a potentially problematic fragment yielding a false positive output that needs to be removed through ligand structure modification. With these properties considered along with their non-toxicity except for Pinocembrin and Naringenin, which exhibited a potential carcinogenic effect through the AMES test (Supplementary Table S6) [32], the following (Table 1) are the molecular structure and molecular weight of the compounds and positive controls which were used as ligands for the molecular docking simulation [33]. Additionally, we found that naringenin only comes second to pinocembrin when docked to the protein 2P31 (Figure 3C) with pinocembrin having the highest binding energy of −10.85 kcal mol^−1^ (Supplementary Table S7). Pinocembrin’s O_1_ atom contributed to an H bond with Ser 102(A), its O_2_ and O_3_ atoms with Ser 102(B), and its O_4_ atom with Thr 107(B) while having hydrophobic interactions with Glu 99(A and B), Arg 106(A and B), and Phe 103(A and B). These amino acid interactions are necessary to be identified as certain amino acids act as a precursor in some pathways, which lead to oxidation. Arginine and tryptophan are two of these which are involved in the iNOS-mediated production of NO while other precursors such as glutamine, alanine, and leucine activate downstream pathways upregulating NO production hence inducing starvation or reduction of the reserved arginine and tryptophan from these proteins help in the inhibition of NO overproduction [14].

Having naringenin, pinocembrin, and eriodictyol as the top three best-scored ligands for all the target proteins is contradictory to other studies with quercetin being the top-scored ligand when docked with other target proteins such as turkey Hb for hemoglobin-mediated lipid oxidation [34] and xanthine oxidase [35]. However, their binding energies all fall within the range for flavonoids which is −5 to −12 kcal/mol for both the antioxidant and anti-inflammatory properties. This contradiction may suggest that quercetin and the other flavonoids may bind better to specific target proteins and for superoxide dismutase, glutathione peroxidase, catalase-mediated oxidation [11], 5-lipoxygenase signaling [22], and cyclooxygenase-mediated inflammation [24]. The above-mentioned ligands bind the best among the flavonoids found in the plant.

## 5. Conclusions

An ADMET prediction of 23 identified phytochemicals from *C. cujete* L. from different groups (iridoid glycosides, phenyl ethanoids, flavonoids, hydroxy benzoyls and hydroxy cinnamoyls) to narrow down the number of phytochemicals to be used for molecular docking analysis has been carried out. The ADMET prediction has revealed that only some of the flavonoids from *C. cujete* showed the most appropriate ADMET properties in a therapeutic dose without any violations and the least number of alerts. These flavonoids docked strongly with the protein targets related to oxidation reactions compared to the positive control, ascorbic acid, which is an indication that these flavonoids can act as agonists, increasing the target proteins’ activities, which helps to overcome stress related to ROS reduction. On the other hand, only three of these flavonoids—naringenin, eriodictyol, and pinocembrin—docked significantly better with the two target proteins (4L9S and 3LN0) associated with inflammation reactions against the positive control, indomethacin, while only ferulic acid falls short when docked with the remaining target protein, 3V99. The findings from this study support the previous docking studies that showed flavonoids to be promising docking ligands to antioxidant and anti-inflammatory protein targets. These results may serve as a reference for further in vitro and in vivo studies exploring the antioxidant and anti-inflammatory activities with the protein targets used in this study to possibly provide potential avenues for the development of a multi-target drug from *C. cujete*.

## Figures and Tables

**Table 1:** Molecular Docking Top scored ligand-protein complex data for receptors 1CB4, 2CAG and 2P31.

	1CB4		2CAG		2P31	
Ligand	Naringenin	Ascorbic Acid	Naringenin	Ascorbic Acid	Pinocembrin	Ascorbic Acid
binding energy	−10.32	−3.46	−11.87	−3.64	−10.85	−4.49
ligand efficiency	−0.52	−0.31	−0.59	−0.33	−0.57	−0.41
inhib constant	27.26	2.93	1.99	2.16	11.08	512.61
inhib constant units	nM	mM	nM	mM	nM	uM
intermol energy	−11.21	−4.65	−12.77	−5.13	−11.45	−5.68
vdw hb desolv energy	−11.21	−4.42	−12.77	−4.93	−11.45	−5.48
electrostatic energy	0	−0.23	0	−0.2	0	−0.21
total internal	−0.08	−1.67	−0.06	−1.68	−0.3	−1.64
torsional energy	0.89	1.19	0.89	1.49	0.6	1.19
unbound energy	−0.08	−1.67	−0.06	−1.68	−0.3	−1.64
cIRMS	0	0	0	0	0	0
refRMS	69.76	72.15	58.64	63.49	28.98	28.06
rseed1	none	none	none	none	none	none
rseed2	none	none	none	none	none	none
H-bond formed	2	3	2	0	0	2

**Table 2:** Molecular Docking Top scored ligand–protein complex data for receptors 3V99, 4L9S and 3LN0.

	3V99		4L9S		3LN0	
Ligand	Pinocembrin	Indomethacin	Eriodictyol	Indomethacin	Naringenin	Indomethacin
binding energy	−11.29	−8.31	−10.39	−9.47	−11.96	−11.22
ligand efficiency	−0.59	−0.33	−0.49	−0.38	−0.6	−0.45
inhib constant	5.29	804.47	24.39	113.87	1.72	6.01
inhib constant units	nM	nM	nM	nM	nM	nM
intermol energy	−11.89	−9.51	−11.88	−10.67	−12.85	−12.41
vdw hb desolv energy	−11.89	−9.51	−11.88	−10.67	−12.85	−12.41
electrostatic energy	0	0	0	0	0	0
total internal	−0.3	−1.54	14.93	−1.45	−0.08	−1.25
torsional energy	0.6	1.19	1.49	1.19	0.89	1.19
unbound energy	−0.3	−1.54	14.93	−1.45	−0.08	−1.25
cIRMS	0	0	0.25	0	0	0
refRMS	90.94	73.39	30.16	38.62	72.45	90.14
rseed1	none	none	none	none	none	none
rseed2	none	none	none	none	none	none
H-bond formed	2	2	4	4	2	0

**Table 3:** Drugs and ligands used from *C. cujete* for antioxidant and anti-inflammatory properties [29].

Drug/Ligand	Structure	Molecular Weight (g/mol)
Ascorbic Acid	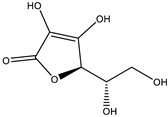	176.12
Indomethacin	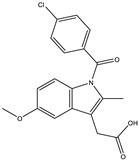	357.79
Luteolin	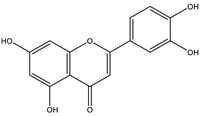	286.24
Eriodictyol	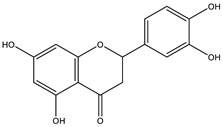	288.25
Pinocembrin	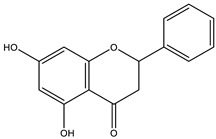	256.25
Naringenin	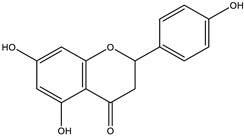	272.25
Quercetin	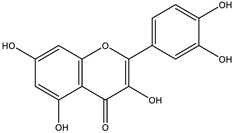	302.24
Apigenin	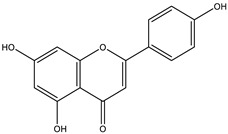	270.24
Ferulic Acid	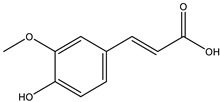	194.18

## Data Availability

Not applicable.

## Supplementary Materials

The following supporting information can be downloaded at: https://www.mdpi.com/article/10.3390/molecules28083547/s1, Table S1: Physicochemical Properties; Table S2: Lipophilicity and water solubility; Table S3: Pharmacokinetics; Table S4: Drug Likeness; Table S5: Medical Chemistry; Table S6: Toxicity; Table S7: Molecular Docking Best scored ligand–protein complex data for all seven ligands with receptors 3V99, 4L9S and 3LN0; Table S8: Molecular Docking Best scored ligand–protein complex data for all seven ligands with receptors 1CB4, 2CAG and 2P31.
