# Supplementary material for: In Silico Analysis of Anti-Inflammatory and Antioxidant Properties of Bioactive Compounds from Crescentia cujete L
Source: Molecules. 2023 Apr 18;28(8):3547. doi: 10.3390/molecules28083547 (PMC10145697; doi:10.3390/molecules28083547)
Supplement: Supplementary file 1 [file molecules-28-03547-s001.zip › molecules-2317849-supplementary.pdf]

Table S1. Physicochemical Properties

Molecule	Luteolin	Eriodictyol	Pinocembrin	Naringenin	Quercetin	Apigenin	Ferulic Acid
Formula	C15H10O6	C15H12O6	C15H12O4	C15H12O5	C15H10O7	C15H10O5	C10H10O4
MW	286.24	288.25	256.25	272.25	302.24	270.24	194.18
#Heavy atoms	21	21	19	20	22	20	14
#Aromatic heavy atoms	16	12	12	12	16	16	6
Fraction Csp3	0	0.13	0.13	0.13	0	0	0.1
#Rotatable bonds	1	1	1	1	1	1	3
#H-bond acceptors	6	6	4	5	7	5	4
#H-bond donors	4	4	2	3	5	3	2
MR	76.01	73.59	69.55	71.57	78.03	73.99	51.63
TPSA	111.13	107.22	66.76	86.99	131.36	90.9	66.76

Table S2. Lipophilicity and water solubility

Molecule	Luteolin	Eriodictyol	Pinocembrin	Naringenin	Quercetin	Apigenin	Ferulic Acid
iLOGP	1.86	1.62	2.11	1.75	1.63	1.89	1.62
XLOGP3	2.53	2.02	2.88	2.52	1.54	3.02	1.51
WLOGP	2.28	1.89	2.48	2.19	1.99	2.58	1.39
MLOGP	-0.03	0.16	1.27	0.71	-0.56	0.52	1
Silicos-IT Log P	2.03	1.56	2.55	2.05	1.54	2.52	1.26
Consensus Log P	1.73	1.45	2.26	1.84	1.23	2.11	1.36
ESOL Log S	-3.71	-3.26	-3.64	-3.49	-3.16	-3.94	-2.11
ESOL Solubility (mg/ml)	5.63E-02	1.60E-01	5.81E-02	8.74E-02	2.11E-01	3.07E-02	1.49E+00
ESOL Solubility (mol/l)	1.97E-04	5.54E-04	2.27E-04	3.21E-04	6.98E-04	1.14E-04	7.68E-03
ESOL Class	Soluble	Soluble	Soluble	Soluble	Soluble	Soluble	Soluble
Ali Log S	-4.51	-3.9	-3.94	-3.99	-3.91	-4.59	-2.52
Ali Solubility (mg/ml)	8.84E-03	3.64E-02	2.93E-02	2.77E-02	3.74E-02	6.88E-03	5.86E-01
Ali Solubility (mol/l)	3.09E-05	1.26E-04	1.14E-04	1.02E-04	1.24E-04	2.55E-05	3.02E-03
Ali Class	Moderately soluble	Soluble	Soluble	Soluble	Soluble	Moderately soluble	Soluble
Silicos-IT LogSw	-3.82	-2.84	-4	-3.42	-3.24	-4.4	-1.42
Silicos-IT Solubility (mg/ml)	4.29E-02	4.19E-01	2.58E-02	1.04E-01	1.73E-01	1.07E-02	7.43E+00
Silicos-IT Solubility (mol/l)	1.50E-04	1.45E-03	1.01E-04	3.82E-04	5.73E-04	3.94E-05	3.83E-02
Silicos-IT class	Soluble	Soluble	Soluble	Soluble	Soluble	Moderately soluble	Soluble

Table S3. Pharmacokinetics

Molecule	Luteolin	Eriodictyol	Pinocembrin	Naringenin	Quercetin	Apigenin	Ferulic Acid
GI absorption	High	High	High	High	High	High	High
BBB permeant	No	No	Yes	No	No	No	Yes
Pgp substrate	No	Yes	No	Yes	No	No	No
CYP1A2 inhibitor	Yes	No	Yes	Yes	Yes	Yes	No
CYP2C19 inhibitor	No	No	Yes	No	No	No	No
CYP2C9 inhibitor	No	No	No	No	No	No	No
CYP2D6 inhibitor	Yes	No	No	No	Yes	Yes	No
CYP3A4 inhibitor	Yes	Yes	No	Yes	Yes	Yes	No
log Kp (cm/s)	-6.25	-6.62	-5.82	-6.17	-7.05	-5.8	-6.41

Table S4. Drug Likeness

Molecule	Luteolin	Eriodictyol	Pinocembrin	Naringenin	Quercetin	Apigenin	Ferulic Acid
Lipinski #violations	0	0	0	0	0	0	0
Ghose #violations	0	0	0	0	0	0	0
Veber #violations	0	0	0	0	0	0	0
Egan #violations	0	0	0	0	0	0	0
Muegge #violations	0	0	0	0	0	0	1
Bioavailability Score	0.55	0.55	0.55	0.55	0.55	0.55	0.85

Table S5. Medical Chemistry

Molecule	Luteolin	Eriodictyol	Pinocembrin	Naringenin	Quercetin	Apigenin	Ferulic Acid
PAINS #alerts	1	1	0	0	1	0	0
Brenk #alerts	1	1	0	0	1	0	1
Leadlikeness #violations	0	0	0	0	0	0	1
Synthetic Accessibility	3.02	3.11	2.96	3.01	3.23	2.96	1.93

Table S6. Toxicity

Molecule	Luteolin	Eriodictyol	Pinocembrin	Naringenin	Quercetin	Apigenin	Ferulic Acid
AMES toxicity	No	No	Yes	Yes	No	No	No
Max. tolerated dose (human)	0.564	-0.239	0.276	0.499	0.775	0.337	0.986
hERG I inhibitor	No	No	No	No	No	No	No
hERG II inhibitor	No	No	No	No	No	No	No
Oral Rat Acute Toxicity (LD50)	2.453	2.257	2.13	2.01	2.607	1.978	2.281
Oral Rat Chronic Toxicity (LOAEL)	1.537	1.855	2.32	2.27	2.464	1.959	1.909
Hepatotoxicity	No	No	No	No	No	No	No
Skin Sensitisation	No	No	No	No	No	No	No
T.Pyriformis toxicity	0.428	0.422	0.882	0.532	0.335	0.517	0.028
Minnow toxicity	1.346	3.052	1.96	1.294	1.957	1.17	2.294

Table S7. Molecular Docking Best scored ligand-protein complex data for all 7 ligands with receptors 3V99, 4L9S and 3LN0

**3V99**	Indomethacin	Luteolin	Eriodictyol	Pinocembrin	Naringenin	Quercetin	Apigenin	Ferulic Acid
binding energy	-8.31	-9.09	-10.02	-11.29	-10.77	-9.38	-9.17	-7.3
ligand efficiency	-0.33	-0.43	-0.48	-0.59	-0.54	-0.43	-0.46	-0.52
inhib constant	804.47	216.67	45.51	5.29	12.7	132.94	189.42	4.48
inhib constant units	nM	nM	nM	nM	nM	nM	nM	uM
intermol energy	-9.51	-10.58	-11.51	-11.89	-11.67	-11.17	-10.36	-9.09
vdw hb desolv energy	-9.51	-10.58	-11.51	-11.89	-11.67	-11.17	-10.36	-9.09
electrostatic energy	0	0	0	0	0	0	0	0
total internal	-1.54	-1.99	15.37	-0.3	-0.1	-2.72	-1.04	17.73
torsional energy	1.19	1.49	1.49	0.6	0.89	1.79	1.19	1.79
unbound energy	-1.54	-1.99	15.37	-0.3	-0.1	-2.72	-1.04	17.73
cIRMS	0	0	0	0	0	0	0	0
refRMS	73.39	46.8	55.1	90.94	89.03	76.25	60.76	45.01
rseed1	none	none	none	none	none	none	none	none
rseed2	none	none	none	none	none	none	none	none
H-bond formed	2	1	0	0	0	2	0	0

**4L9S**	Indomethacin	Luteolin	Eriodictyol	Pinocembrin	Naringenin	Quercetin	Apigenin	Ferulic Acid
binding energy	-9.47	-9.21	-10.39	-9.6	-10.22	-8.86	-8.66	-6.36
ligand efficiency	-0.38	-0.44	-0.49	-0.51	-0.51	-0.4	-0.43	-0.45
inhib constant	113.87	176.91	24.39	92.14	32.19	319.67	448.81	21.67
inhib constant units	nM	nM	nM	nM	nM	nM	nM	uM
intermol energy	-10.67	-10.7	-11.88	-10.19	-11.12	-10.65	-9.85	-8.15
vdw hb desolv energy	-10.67	-10.7	-11.88	-10.19	-11.12	-10.65	-9.85	-8.15
electrostatic energy	0	0	0	0	0	0	0	0
total internal	-1.45	-1.99	14.93	-0.25	-1.98	-2.65	-1.02	17.76
torsional energy	1.19	1.49	1.49	0.6	0.89	1.79	1.19	1.79
unbound energy	-1.45	-1.99	14.93	-0.25	-1.98	-2.65	-1.02	17.76

cIRMS	0	0	0.25	0	0	0	0	0
refRMS	38.62	31.89	30.16	31.28	29.86	31.51	31.92	35.14
rseed1	none	none	none	none	none	none	none	none
rseed2	none	none	none	none	none	none	none	none
H-bond formed	4	2	2	3	2	3	2	2

**3LN0**	Indomethacin	Luteolin	Eriodictyol	Pinocembrin	Naringenin	Quercetin	Apigenin	Ferulic Acid
binding energy	-11.22	-9.82	-11.54	-11.35	-11.96	-6.08	-10.15	-4.72
ligand efficiency	-0.45	-0.47	-0.55	-0.6	-0.6	-0.28	-0.51	-0.34
inhib constant	6.01	63.28	3.46	4.81	1.72	34.73	36.54	345.42
inhib constant units	nM	nM	nM	nM	nM	uM	nM	uM
intermol energy	-12.41	-11.31	-13.03	-11.94	-12.85	-7.87	-11.34	-6.51
vdw hb desolv energy	-12.41	-11.31	-13.03	-11.94	-12.85	-7.82	-11.34	-5.36
electrostatic energy	0	0	0	0	0	-0.05	0	-1.15
total internal	-1.25	-1.98	14.96	-0.3	-0.08	-2.65	-1.02	17.92
torsional energy	1.19	1.49	1.49	0.6	0.89	1.79	1.19	1.79
unbound energy	-1.25	-1.98	14.96	-0.3	-0.08	-2.65	-1.02	17.92
cIRMS	0	0	0	0	0	0	0	0
refRMS	90.14	87.13	92.91	37.4	72.45	81.98	71.73	40.5
rseed1	none	none	none	none	none	none	none	none
rseed2	none	none	none	none	none	none	none	none
H-bond formed	0	1	0	0	0	2	0	0

Table S8. Molecular Docking Best scored ligand-protein complex data for all 7 ligands with receptors 1CB4, 2CAG and 2P31

1CB4	Ascorbic Acid	Luteolin	Eriodictyol	Pinocembrin	Naringenin	Quercetin	Apigenin	Ferulic Acid
binding energy	-3.46	-8.34	-9.34	-9.52	-10.32	-5.29	-8.25	-6.83
ligand efficiency	-0.31	0.4	-0.44	-0.5	-0.52	-0.24	-0.41	-0.49
inhib constant	2.93	764.23	142.3	105.15	27.26	132.82	899.88	9.93
inhib constant units	mM	nM	nM	nM	nM	uM	nM	uM
intermol energy	-4.65	-9.84	-10.83	-10.12	-11.21	-7.08	-9.44	-8.61
vdw hb desolv energy	-4.42	-9.84	-10.83	-10.12	-11.21	-6.96	-9.44	-8.61
electrostatic energy	-0.23	0	0	0	0	-0.12	0	0
total internal	-1.67	-1.98	14.95	-0.29	-0.08	-2.24	-1.02	17.92
torsional energy	1.19	1.49	1.49	0.6	0.89	1.79	1.19	1.79
unbound energy	-1.67	-1.98	14.95	-0.29	-0.08	-2.24	-1.02	17.92
cIRMS	0	0	0	0.09	0	0	0	0
refRMS	72.15	67.01	69.12	69.45	69.76	67.45	67.87	71.4
rseed1	none	none	none	none	none	none	none	none
rseed2	none	none	none	none	none	none	none	none
H-bond formed	3	0	0	0	2	3	0	0

2CAG	Ascorbic Acid	Luteolin	Eriodictyol	Pinocembrin	Naringenin	Quercetin	Apigenin	Ferulic Acid
binding energy	-3.64	-9.33	-11.15	-11.17	-11.87	-9.96	-10.08	-6.76
ligand efficiency	-0.33	-0.44	-0.53	-0.59	-0.59	-0.45	-0.5	-0.48
inhib constant	2.16	145.12	6.68	6.45	1.99	49.71	41.03	11.01

inhib constant units	mM	nM	nM	nM	nM	nM	nM	uM
intermol energy	-5.13	-10.82	-12.64	-11.77	-12.77	-11.75	-11.27	-8.55
vdw hb desolv energy	-4.93	-10.82	-12.64	-11.77	-12.77	-11.75	-11.27	-8.55
electrostatic energy	-0.2	0	0	0	0	0	0	0
total internal	-1.68	-1.98	14.93	-0.3	-0.06	-2.57	-1.03	17.71
torsional energy	1.49	1.49	1.49	0.6	0.89	1.79	1.19	1.79
unbound energy	-1.68	-1.98	14.93	-0.3	-0.06	-2.57	-1.03	17.71
cIRMS	0	0	0	0	0	0	0	0
refRMS	63.49	55.2	58.06	59.93	58.64	53.73	53.26	56.31
rseed1	none	none	none	none	none	none	none	none
rseed2	none	none	none	none	none	none	none	none
H-bond formed	0	1	2	3	2	2	0	0

2P31	Ascorbic Acid	Luteolin	Eriodictyol	Pinocembrin	Naringenin	Quercetin	Apigenin	Ferulic Acid
binding energy	-4.49	-7.19	-9.74	-10.85	-10.16	-6.41	-6.58	-7.15
ligand efficiency	-0.41	-0.34	-0.46	-0.57	-0.51	-0.29	-0.33	-0.51
inhib constant	512.61	5.37	72.43	11.08	35.66	19.86	15.05	5.75
inhib constant units	uM	uM	nM	nM	nM	uM	uM	uM
intermol energy	-5.68	-8.68	-11.23	-11.45	-11.06	-8.2	-7.77	-8.94
vdw hb desolv energy	-5.48	-8.68	-11.23	-11.45	-11.06	-8.2	-7.77	-8.94
electrostatic energy	-0.21	0	0	0	0	0	0	0
total internal	-1.64	-1.91	15.02	-0.3	-0.09	-2.64	-1.02	17.81

torsional energy	1.19	1.49	1.49	0.6	0.89	1.79	1.19	1.79
unbound energy	-1.64	-1.91	15.02	-0.3	-0.09	-2.64	-1.02	17.81
cIRMS	0	0	0	0	0	0	0	0
refRMS	28.06	30.09	30.16	28.98	29.15	27.34	19.82	28.89
rseed1	none	none	none	none	none	none	none	none
rseed2	none	none	none	none	none	none	none	none
H-bond formed	2	0	0	0	0	2	1	1
